# Morphological Manipulation of DNA Gel Microbeads with Biomolecular Stimuli

**DOI:** 10.3390/nano11020293

**Published:** 2021-01-22

**Authors:** Shu Okumura, Benediktus Nixon Hapsianto, Nicolas Lobato-Dauzier, Yuto Ohno, Seiju Benner, Yosuke Torii, Yuuka Tanabe, Kazuki Takada, Alexandre Baccouche, Marie Shinohara, Soo Hyeon Kim, Teruo Fujii, Anthony Genot

**Affiliations:** 1 LIMMS, CNRS-Institute of Industrial Science, UMI 2820, University of Tokyo, Tokyo 153-8505, Japan; shu-ok@iis.u-tokyo.ac.jp (S.O.); lobato@iis.u-tokyo.ac.jp (N.L.-D.); baccouch@iis.u-tokyo.ac.jp (A.B.); shkim@iis.u-tokyo.ac.jp (S.H.K.); tfujii@iis.u-tokyo.ac.jp (T.F.); 2 Department of Bioengineering, The University of Tokyo 7-3-1 Hongo, Bunkyo, Tokyo 113-8654, Japan; nixon@iis.u-tokyo.ac.jp (B.N.H.); marie-s@iis.u-tokyo.ac.jp (M.S.); 3 Institute of Industrial Science, The University of Tokyo 4-6-1 Komaba, Meguro, Tokyo 153-8505, Japan; 4 Department of Chemistry, School of Science, The University of Tokyo 7-3-1 Hongo, Bunkyo, Tokyo 113-8654, Japan; yuto7518@g.ecc.u-tokyo.ac.jp (Y.O.); seiju.benner@gmail.com (S.B.); yuuka923singapore.hongkong@gmail.com (Y.T.); 5 Faculty of Agriculture, The University of Tokyo 7-3-1 Hongo, Bunkyo, Tokyo 113-8654, Japan; ytorii10111@g.ecc.u-tokyo.ac.jp; 6 Faculty of Pharmaceutical Sciences, The University of Tokyo 7-3-1 Hongo, Bunkyo, Tokyo 113-8654, Japan; ktakada427@gmail.com

**Keywords:** DNA nanotechnology, droplet microfluidic device, DNA hydrogels, toehold-strands, dynamic morphological transformation

## Abstract

Hydrogels are essential in many fields ranging from tissue engineering and drug delivery to food sciences or cosmetics. Hydrogels that respond to specific biomolecular stimuli such as DNA, mRNA, miRNA and small molecules are highly desirable from the perspective of medical applications, however interfacing classical hydrogels with nucleic acids is still challenging. Here were demonstrate the generation of microbeads of DNA hydrogels with droplet microfluidic, and their morphological actuation with DNA strands. Using strand displacement and the specificity of DNA base pairing, we selectively dissolved gel beads, and reversibly changed their size on-the-fly with controlled swelling and shrinking. Lastly, we performed a complex computing primitive—A Winner-Takes-All competition between two populations of gel beads. Overall, these results show that strand responsive DNA gels have tantalizing potentials to enhance and expand traditional hydrogels, in particular for applications in sequencing and drug delivery.

## 1. Introduction

Hydrogels are fundamental in bioengineering, where they serve as scaffolds to support and steer the growth of tissues in-vitro, or in drug delivery where they serve as an excipient to carry drugs to target locations [1,2,3,4,5,6]. Hydrogels are also making inroads in single-cell sequencing [7,8]. In this approach, single cells are encapsulated into droplets with hydrogels beads that bear monoclonal DNA barcodes. After lysis in the droplets, the transcripts from each cell are concatenated to their DNA barcodes, and read out with next-generation sequencing—thus enabling transcriptomics at the single-cell level [9].

In that context, so-called stimuli-responsive gels have become an intense focus of research, as engineers seek to gain control over when, where and how gels can form [4,10,11,12,13,14,15,16,17,18]. Gels that respond to temperature or pH are now common—thanks to the availability of pH or temperature sensitive monomers—and are routinely used to control the timing of gelation (for instance to prevent premature gelation). Gels that respond to other physical stimuli like electrical fields or light have also been reported, thanks to the synthesis of photosensitive monomers or by taking advantage of electrophoretic or electroosmotic effects [19,20,21]. By combining photosensitive gels with lithography, one gains spatial control over the structuration of the gel, and for instance one can imprint a gradient of stiffness (which is known to influence the differentiation of tissues) [22,23]. Electrosensitive hydrogels were embedded in soft robots to control their locomotion with external electrical fields [21,24,25]. However, hydrogels that respond to specific biomarkers (e.g., miRNA, and proteins) have been more elusive, although they would be highly useful for a variety of applications—the most obvious being biosensing [26]. In drug delivery, DNA gels that sense biomarkers for cancers could enhance the specificity of delivery of oncogenic drugs to tumors. In single-cell sequencing, gel beads capable of sensing target transcripts could be used to isolate and sequence particular cellular types or states. Yet the monomers that compose common hydrogels [27,28,29,30,31] (polysaccharide, polyacrylamide, alginate, hyaluronic acid, and polyethylene glycol) are small organic molecules that do not interact naturally with arbitrary nucleic acid or proteins.

DNA gels—which gelify by the mutual hybridization of DNA nanostructures into an extended 3D network—have emerged as a new class of hydrogels with ideal properties for stimuli-responsiveness [32,33,34,35]. In addition to being biocompatible and biodegradable, DNA is an ideal material for interfacing hydrogels with the realm of biomolecules. DNA strands are easily conjugated to polymers such as polyacrylamide or polyethylene glycol, and can sense nucleic acids from biological origins (DNA, mRNA, and miRNA), or small molecules and proteins with the help of aptamers. Additionally, DNA nanotechnology now offers a rich toolbox of nanostructures (e.g., DNA origami [36,37,38]) and mechanisms (strand displacement [26]) to control the shape and dynamics of DNA monomers down to the nanoscale. In the past two decades, a rich library of DNA-based gels has been reported [32,33,34,35,39,40,41,42,43,44,45,46,47], along with numerous applications in sequencing, drug delivery, CTC cell capture or qPCR [26,44,45]. DNA gels that are responsive to pH [32,33,34], temperature [35], or enzymes such as CRISPR-Cas12 [48] have been reported in the literature. 

Fabrication of microgels with microfluidics is now well established [49], enabling the generation of spherical microparticles of hydrogels, which are easier to administer in drug delivery [50]. For instance beads of agarose, gelatin or silica were generated with droplets microfluidics [51]. Besides this, research has been done to scale up throughput in view of industrial applications [52,53,54]. For instance, a massively parallel microfluidic device made of Silicon was reported which produces liters of emulsions per hour [52]. 

DNA gels that change their morphology in response to specific DNA stimuli have been reported [43]. They open avenues for drug delivery, where drugs are loaded and released with controlled swelling and shrinking of the hydrogel. However, the morphological changes were often unidirectional, i.e., were not shown to be reversible [43]. And the demonstration was done on macroscopic DNA gels (mm-cm range), although it would be desirable to achieve the same control on microscopic DNA gels, because they would be easier to inject or inhale [55,56,57,58,59,60,61]. Here, we report DNA microgels that morphologically respond to target DNA strands. We prepare batches of monodisperse microbeads of DNA gels with droplet microfluidics, and actuate morphological changes in the beads with strand displacement, a powerful and general mechanism in DNA nanotechnology [62,63,64,65]. We also demonstrate advanced functionalities, such as selective dissolution of gels, or even a Winner-Takes-All algorithm where two populations of beads mutually dissolve each other until only one population is left. The ease and programmability of implementation opens new avenues, not only for drug delivery, but in other domains such as single-cell sequencing or tissue engineering.

## 2. Materials and Methods

### 2.1. General

DNA strands (Table 1) were purchased unpurified from (Integrated DNA Technology, Coralville, IA, USA), except for st1* TAMRA, which was purchased from (Eurofins, Tokyo, Japan). These strands were resuspended in a 1× Tris-EDTA buffer of pH 8.0 (Sigma-Aldrich, St. Louis, MO, USA) and stored at room temperature. The X-motif strands (st1, st2, st3, st4) and their complementary strands (st1*, st2*, st3*, st4*) were resuspended in stock concentrations of 4 mM, while DNA strands with fluorophore (st1FAM and st1*TAMRA) were resuspended in stock concentration of 100 µM. Bridge strand, extender strand and shrinker strands were resuspended in stock concentration of 1 mM.

### 2.2. DNA Hydrogel Concept

**Figure 1:**
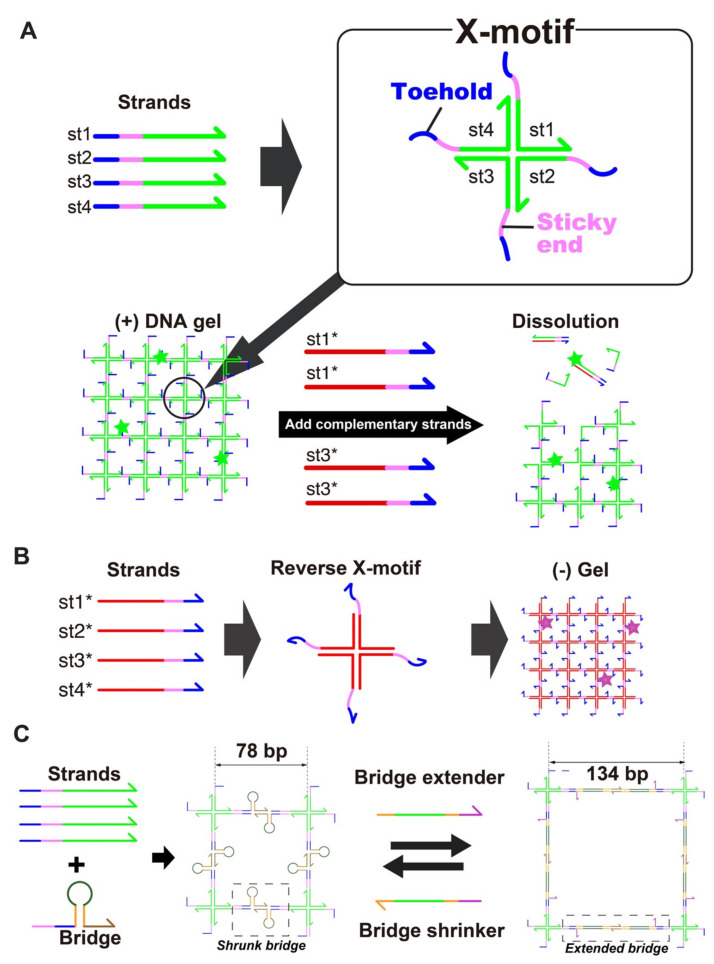
(**A**)Assembly of the DNA hydrogel. The DNA gel forms hierarchically. The four constituent strands (st1, st2, st3, and st4) first mutually bind to form a X shaped motif, a Holliday junction, through the green domains. Each junction exposes 4 sticky-ends (colored pink) by which it binds to other junctions to form a 3D gel. The strands also expose a toehold domain (blue). When a complementary strand (st1*) is added to the solution, this toehold guides and directs the displacement of the target strand (st1), stripping it from the Holliday junction and disrupting the structure. (**B**) Assembly of the (-) DNA hydrogel. Complementary strands of (+) strands also forms a mesh structure, leading to the formation of a gel. (**C**) Controllable swelling and shrinking of DNA gel. A stem-loop structure (a bridge) is installed in the gel structure, bridging two X-motifs. This bridge is extended or shrunk with the addition of extender and shrinker strands, which convert the bridge between a hairpin structure and fully extended duplex structure.

The DNA hydrogel forms through the hybridization of 4 single stranded DNA (i.e., st1, st2, st3 and st4) into a X-shaped Holliday junction (named X-motif) [66]. The X-motif presents 4 identical palindromic sticky ends, by which it binds to other X-motifs to form a 3D gel (Figure 1A). To control the X-motif, we installed a toehold domain at the 5′-end of each strand [62]. The toehold is a short stretch of DNA that gives the complementary strands (st1* to st4*) a thermodynamic advantage to displace individual strands and disrupt the X-motif. Compared to other gels, which may require chemical activation or complex processing for their synthesis, the preparation of the DNA hydrogel is exceedingly simple. It is prepared at room temperature by simply mixing the 4 constituent strands of the X-motif in a saline buffer. To visualize the DNA hydrogel, the DNA hydrogel strand mixture is supplemented with st1 strands with a fluorescent dye at their 3′-end (i.e., st1FAM).

We also prepared two population of gel beads. One population of gel beads is made of (+) strands st1, st2, st3 and st4 (later referred to as the “(+) gels”). The other population is made of the exact complementary (-) strands st1*, st2*, st3* and st4* (referred to as the “(-) gels”). Since the complementary (-) strands have the same mutual connectivity as the native (+) strands, they also form Holliday junctions that gelify together (Figure 1B).

We dynamically reconfigured the gels with DNA strands that triggered reversible swelling and shrinking (Figure 1C). Such morphological changes are employed in drug delivery to control the capture and release of drug payloads by hydrogels [67]. However, they often rely on ionic effects (most often changes in pH) which are nonspecific and difficult to link to particular cellular states. For DNA swelling using extender, the opening of a hairpin loop [43] and elongation with hybridization chain reaction (HCR) [68] have been reported, but these swellings have not been shown to be reversible. Here, we designed a reversible swelling-shrinking system. We installed a hairpin bridge—A motif that connects to the native sticky ends of the gel, while exposing its own sticky ends (Figure 1C). Two bridges mutually bind to each other by their own sticky ends, connecting X-motifs together and driving their gelation. Importantly, the bridge is extended or shrunk with external strands—which in principle can be of arbitrary sequence. In the shrunk state, the bridge adopts a hairpin structure, and the nominal distance between two X-motif is about ~26 nm (78 bp). Upon addition of the extender strand, the loop of the hairpin opens up and becomes a linear duplex. This extends the nominal distance between two X-motif to about ~45 nm (134 bp), a relative extension of ~70%. The extender strand is removed by a shrinker strand, which binds to the extender through a dedicated toehold and zips it off from the bridge. This returns the bridge to its native hairpin structure, thus reducing again the distance between X motifs. 

### 2.3. Cooling Curves of DNA Hydrogel

We mixed the reagents as follows: 1× Tris-EDTA buffer pH 8.0, 150 mM NaCl, 1× Evagreen, and 100 μM of each strand. The solutions were put into a 96-well plate and fluorescently monitored with a CFX96 thermocycler (BioRad, Hercules, CA, USA). Starting from a short (3 min) incubation at 95 °C, the sample was cooled from 95 °C to 20 °C with a cooling rate of 0.1 °C/min.

### 2.4. Beads Preparation with Microfluidic Device

**Figure 2:**
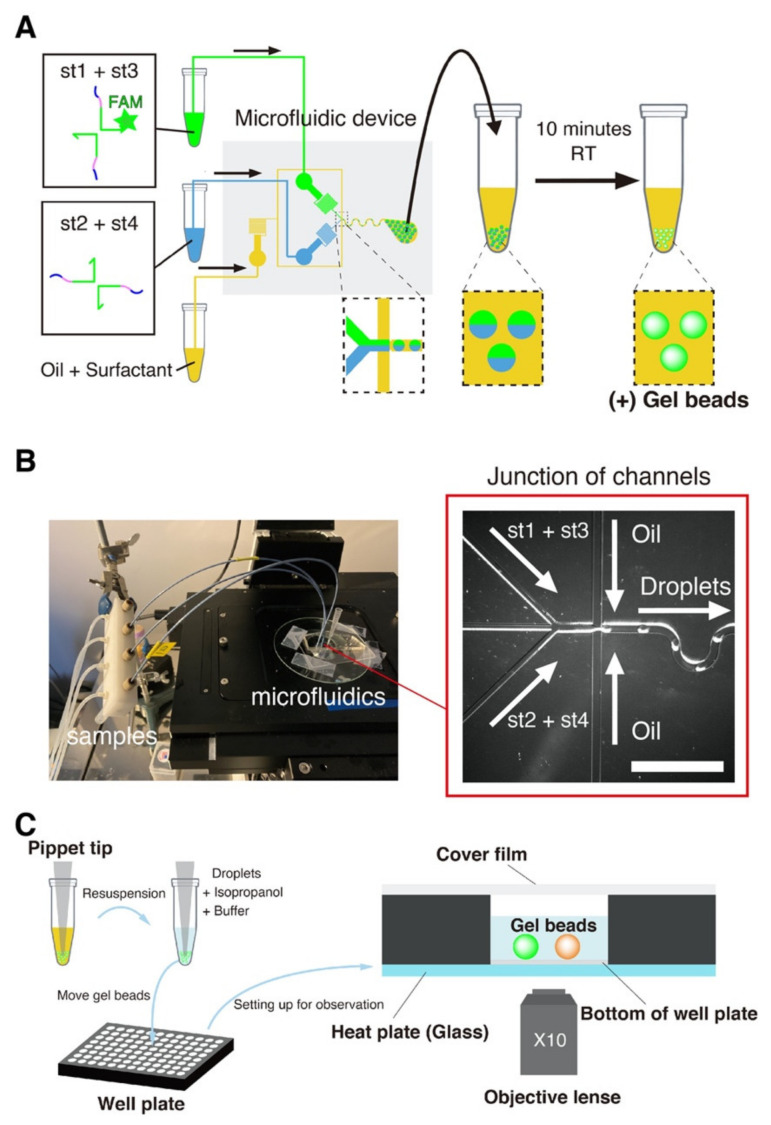
The microfluidic platform for gel beads generation. (**A**) The four strands forming the DNA gel were split into two channels to prevent premature gelation in the device. The two channels meet immediately before a flow-focusing junction, which intersects the aqueous channel with an oil channel. Monodisperse droplets of water in oil are generated at the exit of the junction. After collection, the emulsion was left at room temperature for at least 10 min to allow the DNA strands to gelify. (**B**) Image of the microfluidic platform setup (left). The samples are connected to the device with PEEK tubes directly planted in PDMS. A tip at the outlets collects the water-in-oil emulsion, which creams at top of the tip due to the density of the oil phase. The blowup (right, bright field) shows the junction where droplets are generated. The scale bar is 150 µm. (**C**) Resuspension of the DNA gel beads. After incubation at room temperature, the oil phase is removed and the beads are resuspended in an aqueous buffer. Beads are extracted by mixing them with isopropanol and buffer, and then resuspending them in a larger volume of buffer in a well. The 96-well plate is mounted onto a microscope stage with a transparent glass heat plate for observation.

We produced microbeads of DNA gels with a previously reported droplet microfluidic device (Figure 2A,B and [69,70]). Briefly, an aqueous channel and an oil channel are brought together in the flow-focusing junction of a PDMS microfluidic device [71]. The intersection of these two immiscible fluids generates monodisperse droplets of water in oil, whose size is imposed by the geometry of the junction and the pressures of the channels. The height of the channels is around 50 μm, and this microfluidic device produce droplets with a typical size of 30–60 μm depending on the applied pressures [69]. The length of the pre-injector channel upstream of the junction is 300 μm, which allows visualizing the flows while minimizing residence times [69]. Based on previous estimates, the DNA strands spend several hundreds of milliseconds in the pre-injector area of the chip [2], which is not long enough for the DNA strands to significantly gelify in the device.

To further avoid gelation of the DNA strands before their encapsulation in droplets (which also causes hydrodynamic instability in the mixing channel), we separated strands into distinct aqueous channels that mix just before meeting the junction. 

We started the experiment by preparing two tubes with the DNA strands. The concentration of unmodified strands was 80 μM, the concentration of FAM-labelled st1 was 20 μM, and the concentration of TAMRA-labelled st1* was 2 μM. (The gelation concentration was chosen based on a previous a report on the gelation of X-motifs. [72]. It ensured that the gels were sufficiently stiff and could be handled easily and without breaking). We prepared a tube with Mineral Oil (Sigma-Aldrich, St. Louis, MO, USA) supplemented with 2% *w*/*w* of surfactant Span 80 (TCI, Japan) for stabilizing the droplets [73,74]. After preparing these tubes, we connected them to the inlets of a PDMS device with PEEK tubes. We operated the device by pressuring the tubes with a nitrogen-based pressure controller (MFCS-EZ, Fluigent, Le Kremlin-Bicêtre, France)—which works in a pressure range of 50–800 mbar. The microfluidic device was operated at room temperature. The device was operated as follows. We first set the oil pressure to ~800 mbar, which is about the maximum possible pressure at which operation is stable for our controller and device. We then quickly increased the pressure of the aqueous solution from 0 to about 100 mbar, adjusting it slightly so as to obtain droplets with a diameter of 50 μm. The resulting throughput of droplets generation was typically of several microliters per minute. After the droplets were collected in a pipette tip planted at the outlet, and we waited at least 10 min droplets, after which they are stable at room temperature for extended periods of time (at least a month).

### 2.5. Beads Assay

In order to observe the morphology of the DNA beads, we resuspended them in an aqueous buffer (made by adding 150 mM NaCl to 1× Tris-EDTA buffer pH 8.0, and then vortexing for 30 s). (Note that in the present study, “droplets” refer to vesicles of water in oil, while “beads” refer to spherically-shaped microgels of DNA strands). We observed the gel beads in microwell plates (PerkinElmer, Waltham, MA, USA) with epifluorescence microscopy (Ti-2, Nikon, Tokyo, Japan) using a 10× objective (Nikon, Tokyo, Japan) and appropriate filters (Figure 2C). To remove the oil phase, we first mixed the emulsion coming out of the device with isopropanol in a buffer (1μL of isopropanol, 1μL of droplet, and 8 μL of buffer). After that, we put this solution in the well plate and filled it up to 50 μL with a buffer. In order to avoid bias in the number of gel beads between samples, the tube contain hydrogel beads was well stirred before transfer of the beads to the well plate. Furthermore, we closed the well with a PCR film (Biorad, Hercules, CA, USA) to prevent evaporation of the solution.

### 2.6. Selective Dissolution

Solution of 1 μL of (+) droplets and 1 μL of (-) droplets were resuspended and mixed in 50 μL of buffer. After leaving them 10 min to sit at the bottom of the well plate,1 μL of 1 mM st1 and 1 μL of 1 mM st3 were added to selectively dissolve the (-) gel beads. The process was carried out at room temperature and observed by fluorescence microscopy.

### 2.7. Winner-Takes-All Function

5 μL of (+) droplets and 5 μL of (-) droplets were resuspended into separate tubes with 20 μL of buffer. The contents of the tubes were then mixed with nominally different ratios (100%:0%, 75%:25%, 50%:50%, 25%:75% and 0%:100%) into five tubes. Then each tube was filled up to 50 μL with buffer, and transferred to the well plate. The well plate was heated to 40 °C, and the evolution of the beads was followed by microscopy. 

### 2.8. Swelling and Shrinking 

To insert the bridge strands within the (+) gel beads, 80 μM of st1, st2, st3, st4, and 160 μM of bridge were mixed during the beads generation by droplet microfluidic. After generation of the droplets, they were resuspended in 50 μL of buffer, transferred into 96 well plates with a flat glass bottom. The plate was set onto microscope stage with a transparent glass heater set at 40 °C for observation, and 1 μL of bridge extender at 1 mM was added to trigger the swelling. The observation was paused after 90 min of incubation, and we briefly opened the sealing film to add 2 μL of 1 mM bridge shrinker before resuming the observation for another 90 min. It is important to note that the gel beads moved during injection of the actuating strands, thus the observation for shrinking was made with a different field of view than for swelling. For each time-lapse image, 25 beads were randomly chosen in the region of interest (representing about 1/4 of the actual field of view of the camera) and their size was measured. Outsized beads (i.e., beads that are typically twice larger than the average size) were excluded from this selection. The measurement data were statistically analyzed and plotted using MATLAB.

## 3. Results

### 3.1. Hydrogel Formation

**Figure 3:**
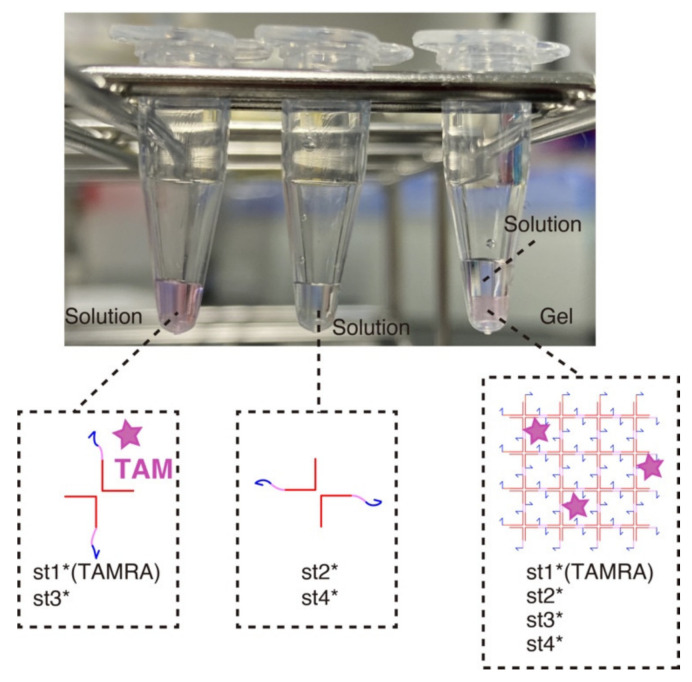
Macroscopic visualization of the DNA hydrogel. Three combinations of strands were mixed in a PCR tube and left at room temperature for about 15 min. The left and middle tubes contain st1*(TAMRA)-st3* strands and st2*-st4* strands, respectively (80 µM for each strand). The strands remained in the solution phase because no complete X-motif monomer can be formed. The right tube contains 20 µL of each of the left two tubes with addition of the buffer. After 15 min of incubation, the assembly of the X-motifs into a 3D gel became visible. The content of the tube was visibly separated into two phase, one solution phase and one gel phase.

We first verified the gelation of the DNA hydrogels at a macroscopic level (Figure 3). Mixing two strands out of four (the strands that are diagonally opposed in the X-motif) does not result in phase separation—one of the hallmarks of gelation—and the solution remains homogeneous. However, when the four strands are mixed together, we observe a clear sol-gel transition with the content of the tube partitioning in two phases: a gel phase at the bottom of the tube (in pink) and a liquid at the top (transparent).

### 3.2. Cooling Curve of DNA Hydrogel

**Figure 4:**
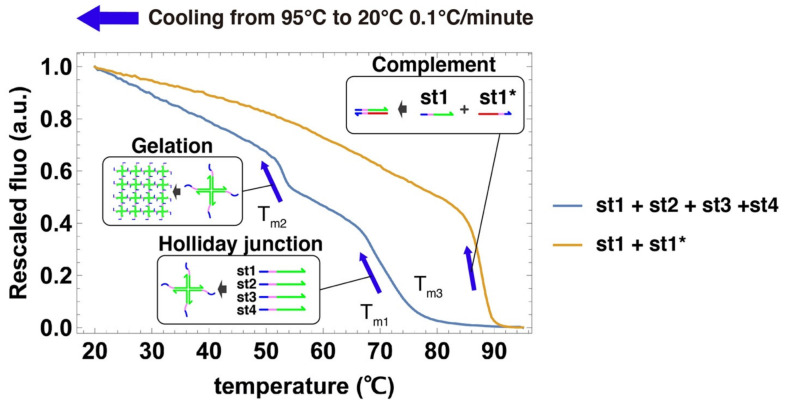
Cooling curves of the components of the DNA gels. DNA strands are mixed in a PCR tube, heated at high temperature, then slowly cooled down. Their hybridization is followed with a DNA intercalant by EvaGreen fluorescence (excitation wavelength is 450–495 nm and emission wavelength is 515–530 nm), which reports on the number of base pairs formed. The blue curve shows the cooling curve of a solution containing all the strands composing the DNA hydrogel. The yellow curve shows the cooling curve of strands st1 and st1*. All strands are present at 100 µM concentration.

We acquired cooling curves of the DNA gels to estimate their characteristic temperatures (Figure 4) [75]. Two large fluorescence jumps were observed during the cooling of the 4 strands that compose the gel. The first jump occurred around T_m1_ = 68 °C, which we assign to the assembly of the 4 strands into a Holliday junction [66,76]. The second jump occurs at T_m2_ = 52 °C, which we assign to the mutual binding of Holliday junctions by their sticky ends into a gel. This temperature T_m2_ gives the upper limit of stability for the gelation process. We also measured the cooling curve for the hybridization of st1 with its complementary strand st1*, a process that we use later to disrupt the Holliday junction and dissolve the gel. As expected, the duplex is more stable (T_m3_ = 87.5 °C) than the Holliday junction because DNA bases are fully paired and there is no entropic linker. This measurement supports the assumption that st1* can thermodynamically drive the disruption of the Holliday junction. 

### 3.3. Selective Dissolution

**Figure 5:**
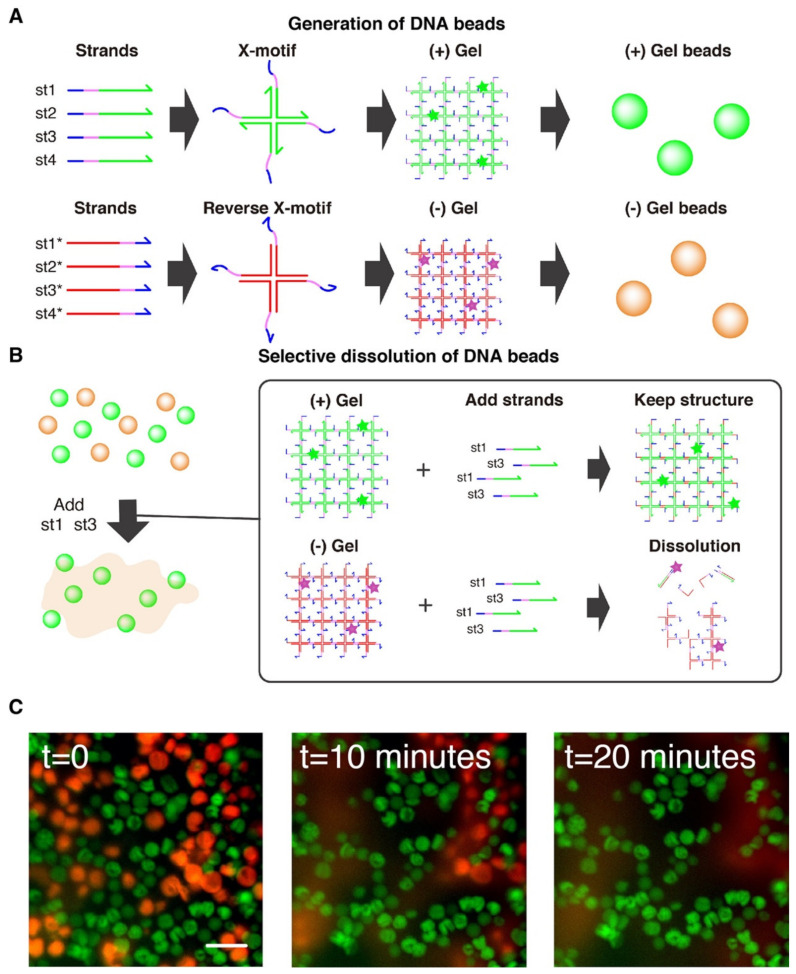
Selective dissolution of DNA gel beads. (**A**) Two populations of gel beads were generated with microfluidic. One population of (+) gel beads is made from strands st1, st2, st3, st4. The (-) population of gel beads is made of the exact complementary strands (st1*, st2*, st3*, st4*), which also form a gel. (**B**) Workflow for selective dissolution. The (+) and (-) population are mixed together, and st1 and st3 are added in large excess. These strands invade the X-motif of the (-) gels through their toehold (blue), causing the disruption of the motif and the dissolution of the gel. (+) gel beads are unaffected because they are inert with respect to st1 and st3. (**C**) Microscopy observation of population of (+) gel beads (in green) and (-) gel beads (in red). The t = 0 image shows the gels just after addition and pipetting of the strand st1 and st3. About 20 min after addition of st1 and st3, the quasi complete dissolution of (-) gel beads can be observed, while the (+) gels remain intact. The scale bar is 80 µm.

We then experimentally demonstrated the generation of gel beads with droplets microfluidics, and their controlled dissolution with specific ssDNA (Figure 5). We prepared the population of (+) gel beads and (-) gels beads with microfluidics, and resuspended them in microwell plates. Microscopy observation confirmed the successful generation of the beads. Although the content of the droplets was resuspended into an aqueous buffer, we could observe two distinct phases: a continuous phase (buffer) and an aggregated phase (+) gel beads in red or (-) gel beads in green. This confirms that gelation was successful in droplets, as otherwise the DNA strands would have dissolved into the aqueous buffer. The size of the beads were 42.0 ± 3.3 μm for (+) gels and 40.0 ± 2.5 μm for (-) gels at room temperature.

We then confirmed the selective dissolution of one population of gel beads while keeping the other population unaffected. To do that, we added a large excess of strands st1 and st3 to the solution. Those strands invade the (-) gel beads, binding to st1* and st3* by their toehold domain to initiate strand displacement that disrupts the X-motif and causes the dissolution of the (-) gel beads (Figure 5B). On the other side, the (+) gel beads remain intact as they do not interact with the strands st1 and st3. This was confirmed experimentally, and we observed the rapid disappearance of (-) gel beads (in red) from the population of beads over the course of 20 min. The speed of dissolution slightly varied from bead to bead, which may reflect local variation in the time needed for the dissolving strands to reach their target. Overall, this shows that DNA gels can be shaped into micrometric beads and sequence-selectively dissolved. 

### 3.4. Winner-Takes-All

We then exploited the complementary of (+) and (-) gel beads to “compare” their concentrations with a mechanism of mutual dissolution, implementing a Winner-Take-All computation. We start with two populations of (+) and (-) gel beads mixed in an unequal ratio, and we get them to mutually dissolve each other, until only one population of beads is left. This kind of Winner-Takes-All mechanism is highly nonlinear, and has been used to devise DNA-based classifiers [77]. Since (+) and (-) gel beads are perfectly complementary, they thermodynamic product is a solution containing fully paired duplexes (e.g., st1 and st1*), which are more thermodynamically stable than the gel structures (Figure 4). However this process is slow at room temperature, and we only observe the kinetic products (separate populations of (+) and (-) gel beads).

We reasoned that heating would “loosen up” the gel structures, partially breaking some sticky ends, unwinding some arms of the X-motif. If this happens when two (+) and (-) gel beads are close to each other, then complementary strands belonging to separate beads (e.g., st1 and st1*) could start to hybridize and drive the resolution of the Holliday junction into fully paired duplexes. This resolution is heavily favored thermodynamically for two reasons: the final duplexes have more base-pairing than the X-motif, and they lack the flexible linkers of the X-motif, which have an entropic cost. Since this process is thermodynamically downhill, it only needs to overcome small kinetics barriers. We chose to heat up the solution of 40 °C, which is halfway between room temperature and the gelation temperature (~50 °C).

**Figure 6:**
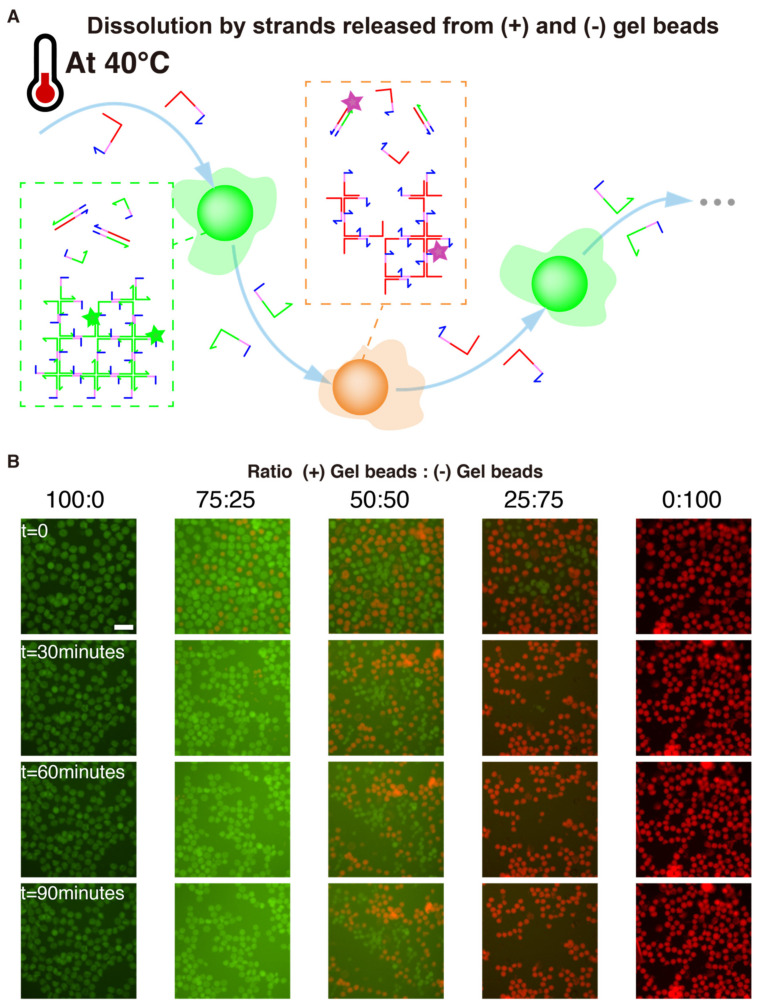
Winner-Takes-All competition between gel beads. (**A**) (+) and (-) gel beads are perfectly complementary, and should mutually “dissolve” each other to leave only fully paired duplexes. However, this process is slow at room temperature. We hypothesized that heating the solution would catalyze this thermodynamically downhill process. Once a strand is peeled from a X-motif, this exposes more single-stranded domains which can react with their complement in the other population of gels. (**B**) Fluorescence time-lapse of gel beads and (-) gel beads mixed in various ratios at 40 °C. The scale bar is 150 µm.

We experimentally validated those assumptions with 5 ratios of (+) and (-) gel beads (Figure 6). For a 3-fold excess of one population over the other (e.g., 75:25 or 25:75), we observed the complete dissolution of the minority population. This mutual dissolution was relatively fast, occurring over a timescale of dozens of minutes. As a control, we also confirmed that dissolution did not occur for 100:0 and 0:100 mixing ratios, which indicates that dissolution was not caused by heating itself, but rather by direct interactions between (+) and (-) beads. We also tried an equal distribution 50:50 of (+) and (-) beads. In this case both populations shrank, but without leaving a clear winner. We hypothesize that a large excess of one population over the other is needed to fully drive the dissolution because the process is not catalytic. 

### 3.5. Swelling and Shrinking of Gels

**Figure 7:**
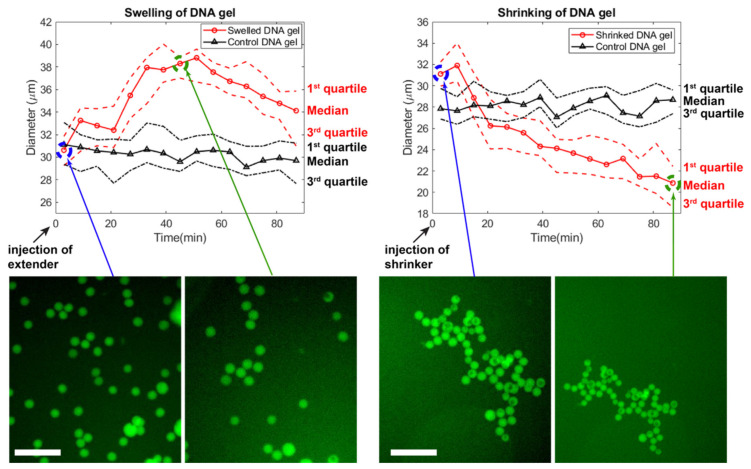
Time-course of the swelling and shrinking of DNA gels. Extender or shrinker strands are added at the beginning, and the distribution of beads’ diameter is followed by microscopy. For each frame, 25 beads were chosen at random in the field of view, excluding large outlier beads. After 90 min, the shrinker strand was injected and beads in a different field of view were followed. The scale bar is 150 µm.

We experimentally tested the swelling and shirking of gel beads (Figure 7). Starting from gel beads comprising bridges (median diameter of 30 μm), we added extender strands to the well. The gel beads swelled up by ~30% in ~50 min, reaching a median diameter of ~38 μm (Video S1). During this time, the size of beads in the control well remained stable. After about 50 min, the beads slightly shrunk spontaneously, for reasons that are unclear. The magnitude of swelling is also lesser than expected, which may be due to various reasons. (Bridges compete with the native sticky ends during gelation, and may not be completely inserted in the matrix. Or the extending strands may not be able to diffuse fully inside the gel matrix, limiting the number of bridges which are extended.) In any case, the speed and magnitude of swelling is consistent with other reports using strand displacement to actuate DNA gels [43,78]. We then demonstrated shrinking of the DNA gel beads by adding remover strands that peel off the extended from the bridge. The median diameter shrunk from ~31 μm to ~20 μm over the course of ~80 min (Video S2). Overall, the reversible shrinking and swelling suggest that our DNA hydrogel design worked as expected, and could find applications in drug delivery.

## 4. Conclusions

We demonstrated the generation of DNA gel beads, as well as their controlled swelling, shrinking and dissolution. This degree of control over the gel—afforded by the programmability of DNA base pairing—could find direct applications in drug delivery. A drug payload could be entrapped in the mesh of a gel bead, and selectively released in presence of RNA biomarkers at specific sites in the human body [79,80,81]. At the moment the mesh (~26 nm) is probably too large to accommodate antibodies (~10 nm) [82], but the arms of the X-motif could in principle be truncated to size the mesh down. Antibodies could also be conjugated to a DNA strand (a routine operation) and directly coupled to the gel matrix with anchoring strands [83,84,85]. Further work in that direction should also find mechanisms to reduce the concentration of signal strands needed to trigger dissolution of the gel. Catalytic mechanisms based on strand-displacement [62,63] could in principle drive this process. 

Programmable DNA gels could also find applications in single-cell sequencing (where gel beads and droplet microfluidics are already routine) for instance to extract from a cellular the transcripts belonging to a specific cellular type [9]. In tissue engineering, DNA gel beads could support complex engineering of spheroids [86], for instance to spatially organize multicellular spheroid, to select spheroid with given phenotypes, or to massively screen culture conditions [87,88]. Lastly, it could also find application at the intersection of regenerative medicine and drug delivery. The packing of hydrogel microparticles at the site of an injury has been proposed as a way to heal tissues. DNA gel beads could support this process, with the added benefit of a stimuli-sensitive control of their morphology, which could help to guide the healing of injuries.

## Figures and Tables

**Table 1:** Sequences of DNA strands used in this study.

st1	5′-**CACTCTTCTGGATCCAGTTTGTTATCGCAGGAGCGTCGGTATTCAAA**-3′
st2	5′-**CACTCTTCTGGATCCAGTTTGAATACCGACGCCACGACCTAATCTTA**-3′
st3	5′-**CACTCTTCTGGATCCAGTAAGATTAGGTCGTGATGGTGAAATGTAAA**-3′
st4	5′-**CACTCTTCTGGATCCAGTTTACATTTCACCATTCCTGCGATAACAAA**-3′
st1*	5′-**TTTGAATACCGACGCTCCTGCGATAACAAACTGGATCCAGAAGAGTG**-3′
st2*	5′-**TAAGATTAGGTCGTGGCGTCGGTATTCAAACTGGATCCAGAAGAGTG**-3′
st3*	5′-**TTTACATTTCACCATCACGACCTAATCTTACTGGATCCAGAAGAGTG**-3′
st4*	5′-**TTTGTTATCGCAGGAATGGTGAAATGTAAACTGGATCCAGAAGAGTG**-3′
st1 [FAM]	5′-**CTGGATCCAGTTTGTTATCGCAGGAGCGTCGGTATTCAAA**[FAM]-3′
st1* [TAM]	5′-**TTTGAATACCGACGCTCCTGCGATAACAAACTGGATCCAG**[TAM]-3′
Bridge	5′-C**TGGATCCAGAAGAGTGTACACTCACTCAGATGAGACAGTGAGTGTAATGCTAGCAT**-3′
Bridge extender	5′-**AGTGAGCTGTCTCATCTGAGTGAGTGTA**-3′
Bridge shrinker	5′-**TACACTCACTCAGATGAGACAGCTCACT**-3′

## Data Availability

The data presented in this study are available on request from the corresponding author.

## Supplementary Materials

The following are available online at https://www.mdpi.com/2079-4991/11/2/293/s1, Video S1: Swelling of DNA hydrogels. Video S2: Shrinking of DNA hydrogels.

## References

[1] Bulpitt P., Aeschlimann D. (1999). New Strategy for Chemical Modification of Hyaluronic Acid: Preparation of Functionalized Derivatives and Their Use in the Formation of Novel Biocompatible Hydrogels. J. Biomed. Mater. Res..

[2] Molinaro G., Leroux J., Damas J., Adam A. (2002). Biocompatibility of thermosensitive chitosan-based hydrogels: An in vivo experimental approach to injectable biomaterials. Biomaterials.

[3] Lee K.Y., Mooney D.J. (2001). Hydrogels for Tissue Engineering. Chem. Rev..

[4] Liu S., Wang P., Huang G., Wang L., Zhou J., Lu T.J., Xu F., Lin M. (2015). Reaction-induced swelling of ionic gels. Soft Matter.

[5] Zhao J., Zhao X., Guo B., Ma P.X. (2014). Multifunctional Interpenetrating Polymer Network Hydrogels Based on Methacrylated Alginate for the Delivery of Small Molecule Drugs and Sustained Release of Protein. Biomacromolecules.

[6] Hotta R., Cheng L.S., Graham H.K., Nagy N., Belkind-Gerson J., Mattheolabakis G., Amiji M.M., Goldstein A.M. (2016). Delivery of enteric neural progenitors with 5-HT4 agonist-loaded nanoparticles and thermosensitive hydrogel enhances cell proliferation and differentiation following transplantation in vivo. Biomaterials.

[7] Wang X., Allen W.E., Wright M., Sylwestrak E.L., Samusik N., Vesuna S., Evans K., Liu C., Ramakrishnan C., Liu J. (2018). Three-dimensional intact-tissue sequencing of single-cell transcriptional states. Science.

[8] Zheng G.X.Y., Terry J.M., Belgrader P., Ryvkin P., Bent Z.W., Wilson R., Ziraldo S.B., Wheeler T.D., McDermott G.P., Zhu J. (2017). Massively parallel digital transcriptional profiling of single cells. Nat. Commun..

[9] Zilionis R., Nainys J., Veres A., Savova V., Zemmour D., Klein A.M., Mazutis L. (2017). Single-cell barcoding and sequencing using droplet microfluidics. Nat. Protoc..

[10] Kim Y.S., Liu M., Ishida Y., Ebina Y., Osada M., Sasaki T., Hansen J.C., Takata M., Aida T. (2015). Thermoresponsive actuation enabled by permittivity switching in an electrostatically anisotropic hydrogel. Nat. Mater..

[11] Shi Q., Liu H., Tang D., Li Y., Li X., Xu F. (2019). Bioactuators based on stimulus-responsive hydrogels and their emerging biomedical applications. NPG Asia Mater..

[12] Chen J.-P., Cheng T.-H. (2006). Thermo-Responsive Chitosan-graft-poly(N-isopropylacrylamide) Injectable Hydrogel for Cultivation of Chondrocytes and Meniscus Cells. Macromol. Biosci..

[13] Beebe D.J., Moore J.S., Bauer J.M., Yu Q., Liu R.H., Devadoss C., Jo B.-H. (2000). Functional hydrogel structures for autonomous flow control inside microfluidic channels. Nat. Cell Biol..

[14] Nakagawa H., Hara Y., Maeda S., Hashimoto S. (2011). A Pendulum-Like Motion of Nanofiber Gel Actuator Synchronized with External Periodic pH Oscillation. Polymers.

[15] Yoshida R., Uchida K., Kaneko Y., Sakai K., Kikuchi A., Sakurai Y., Okano T. (1995). Comb-type grafted hydrogels with rapid deswelling response to temperature changes. Nat. Cell Biol..

[16] Hao L., Yegin C., Talari J.V., Oh J.K., Zhang M., Sari M.M., Zhang L., Min Y., Akbulut M., Bin Jiang B. (2018). Thermo-responsive gels based on supramolecular assembly of an amidoamine and citric acid. Soft Matter.

[17] Zhong R., Xiao M., Zhu C., Shen X., Tang Q., Zhang W., Wang L., Song S., Qu X., Pei H. (2018). Logic Catalytic Interconversion of G-Molecular Hydrogel. ACS Appl. Mater. Interfaces.

[18] Zhong R., Tang Q., Wang S., Zhang H., Zhang F., Xiao M., Man T., Qu X., Li L., Zhang W. (2018). Self-Assembly of Enzyme-Like Nanofibrous G-Molecular Hydrogel for Printed Flexible Electrochemical Sensors. Adv. Mater..

[19] Kim S.J., Kim H.I., Park S.J., Kim I.Y., Lee S.H., Lee T.S.I., Kim S. (2005). Behavior in electric fields of smart hydrogels with potential application as bio-inspired actuators. Smart Mater. Struct..

[20] Wang E., Desai M.S., Lee S.-W. (2013). Light-Controlled Graphene-Elastin Composite Hydrogel Actuators. Nano Lett..

[21] Keplinger C., Sun J.-Y., Foo C.C., Rothemund P., Whitesides G.M., Suo Z. (2013). Stretchable, Transparent, Ionic Conductors. Science.

[22] Kidoaki S., Matsuda T. (2008). Microelastic gradient gelatinous gels to induce cellular mechanotaxis. J. Biotechnol..

[23] Kawano T., Kidoaki S. (2011). Elasticity boundary conditions required for cell mechanotaxis on microelastically-patterned gels. Biomaterials.

[24] Kang Y.-W., Woo J., Lee H.-R., Sun J.-Y. (2019). A mechanically enhanced electroactive hydrogel for 3D printing using a multileg long chain crosslinker. Smart Mater. Struct..

[25] Cvetkovic C., Raman R., Chan V., Williams B.J., Tolish M., Bajaj P., Sakar M.S., Asada H.H., Saif M.T.A., Bashir R. (2014). Three-dimensionally printed biological machines powered by skeletal muscle. Proc. Natl. Acad. Sci. USA.

[26] Zhao Y., Zhao X., Tang B., Xu W., Li J., Hu J., Gu Z. (2010). Quantum-Dot-Tagged Bioresponsive Hydrogel Suspension Array for Multiplex Label-Free DNA Detection. Adv. Funct. Mater..

[27] Oliveira J., Reis R. (2008). Hydrogels from polysaccharide-based materials: Fundamentals and applications in regenerative medicine. Natural-Based Polymers for Biomedical Applications.

[28] Sun J., Tan H. (2013). Alginate-Based Biomaterials for Regenerative Medicine Applications. Materials.

[29] Bai B., Zhou J., Yin M. (2015). A comprehensive review of polyacrylamide polymer gels for conformance control. Pet. Explor. Dev..

[30] Xu X., Jha A.K., Harrington D.A., Farach-Carson M.C., Jia X. (2012). Hyaluronic acid-based hydrogels: From a natural polysaccharide to complex networks. Soft Matter.

[31] Shibayama M., Li X., Sakai T. (2019). Precision polymer network science with tetra-PEG gels—A decade history and future. Colloid Polym. Sci..

[32] Guo W., Lu C.-H., Qi X.-J., Orbach R., Fadeev M., Yang H.-H., Willner I. (2014). Switchable Bifunctional Stimuli-Triggered Poly-N-Isopropylacrylamide/DNA Hydrogels. Angew. Chem. Int. Ed..

[33] Cheng E., Xing Y., Chen P., Yang Y., Sun Y., Zhou D., Xu L., Fan Q., Liu D. (2009). A pH-Triggered, Fast-Responding DNA Hydrogel. Angew. Chem. Int. Ed..

[34] Guo W., Lu C.-H., Orbach R., Wang F., Qi X.-J., Cecconello A., Seliktar D., Willner I. (2015). pH-Stimulated DNA Hydrogels Exhibiting Shape-Memory Properties. Adv. Mater..

[35] Xing Y., Cheng E., Yang Y., Chen P., Zhang T., Sun Y., Yang Z., Liu D. (2010). Self-Assembled DNA Hydrogels with Designable Thermal and Enzymatic Responsiveness. Adv. Mater..

[36] Rothemund P.W.K. (2006). Folding DNA to create nanoscale shapes and patterns. Nat. Cell Biol..

[37] Douglas S.M., Dietz H., Liedl T., Högberg B., Graf F., Shih W.M. (2009). Self-assembly of DNA into nanoscale three-dimensional shapes. Nat. Cell Biol..

[38] Han D., Pal S., Nangreave J., Deng Z., Liu Y., Yan H. (2011). DNA Origami with Complex Curvatures in Three-Dimensional Space. Science.

[39] Sato Y., Sakamoto T., Takinoue M. (2020). Sequence-based engineering of dynamic functions of micrometer-sized DNA droplets. Sci. Adv..

[40] Frank-Kamenetskiĭ M.D., Anshelevich V.V., Lukashin A.V. (1987). Polyelectrolyte model of DNA. Sov. Phys. Uspekhi.

[41] Um S.H., Lee J.B., Park N., Kwon S.Y., Umbach C.C., Luo D. (2006). Enzyme-catalysed assembly of DNA hydrogel. Nat. Mater..

[42] Gehring K., Leroy J.-L., Guéron M. (1993). A tetrameric DNA structure with protonated cytosine-cytosine base pairs. Nat. Cell Biol..

[43] Murakami Y., Maeda M. (2005). DNA-Responsive Hydrogels That Can Shrink or Swell. Biomacromolecules.

[44] Song P., Ye D., Zuo X., Li J., Wang J., Liu H., Hwang M.T., Chao J., Su S., Wang L. (2017). DNA Hydrogel with Aptamer-Toehold-Based Recognition, Cloaking, and Decloaking of Circulating Tumor Cells for Live Cell Analysis. Nano Lett..

[45] Choi W., Yeom S.Y., Kim J., Jung S., Jung S., Shim T.S., Kim S.K., Kang J.Y., Lee S.H., Cho I.-J. (2018). Hydrogel micropost-based qPCR for multiplex detection of miRNAs associated with Alzheimer’s disease. Biosens. Bioelectron..

[46] Hartman M.R., Yang D., Tran T.N.N., Lee K., Kahn J.S., Kiatwuthinon P., Yancey K.G., Trotsenko O., Minko S., Luo D. (2013). Thermostable Branched DNA Nanostructures as Modular Primers for Polymerase Chain Reaction. Angew. Chem. Int. Ed..

[47] Li C., Faulkner-Jones A., Dun A.R., Jin J., Chen P., Xing Y., Yang Z., Li Z., Shu W., Liu D. (2015). Rapid Formation of a Supramolecular Polypeptide-DNA Hydrogel for In Situ Three-Dimensional Multilayer Bioprinting. Angew. Chem. Int. Ed..

[48] English M.A., Soenksen L.R., Gayet R.V., de Puig H., Angenent-Mari N.M., Mao A.S., Nguyen P.Q., Collins J.J. (2019). Programmable CRISPR-responsive smart materials. Science.

[49] Xu S., Nie Z., Seo M., Lewis P., Kumacheva E., Stone H.A., Garstecki P., Weibel D.B., Gitlin I., Whitesides G.M. (2005). Generation of Monodisperse Particles by Using Microfluidics: Control over Size, Shape, and Composition. Angew. Chem..

[50] Daly A.C., Riley L., Segura T., Burdick J.A. (2020). Hydrogel microparticles for biomedical applications. Nat. Rev. Mater..

[51] Desbois L., Padirac A., Kaneda S., Genot A.J., Rondelez Y., Hober D., Collard D., Fujii T. (2012). A microfluidic device for on-chip agarose microbead generation with ultralow reagent consumption. Biomicrofluidics.

[52] Yadavali S., Jeong H.-H., Lee S.H., Issadore D. (2018). Silicon and glass very large-scale microfluidic droplet integration for terascale generation of polymer microparticles. Nat. Commun..

[53] Yelleswarapu V., Buser J.R., Haber M., Baron J., Inapuri E., Issadore D. (2019). Mobile platform for rapid sub–picogram-per-milliliter, multiplexed, digital droplet detection of proteins. Proc. Natl. Acad. Sci. USA.

[54] de Rutte J.M., di Carlo D., di Carlo D. (2019). Scalable High-Throughput Production of Modular Microgels for In Situ Assembly of Microporous Tissue Scaffolds. Adv. Funct. Mater..

[55] Mealy J.E., Chung J.J., Jeong H.-H., Issadore D., Lee S.H., Atluri P., Burdick J.A. (2018). Injectable Granular Hydrogels with Multifunctional Properties for Biomedical Applications. Adv. Mater..

[56] Caldwell A.S., Campbell G.T., Shekiro K.M.T., Anseth K.S. (2017). Clickable Microgel Scaffolds as Platforms for 3D Cell Encapsulation. Adv. Healthc. Mater..

[57] Griffin D.R., Weaver W.M., Scumpia P.O., di Carlo D., Segura T. (2015). Accelerated wound healing by injectable microporous gel scaffolds assembled from annealed building blocks. Nat. Mater..

[58] Wade R.J., Bassin E.J., Rodell C.B., Burdick J.A. (2015). Protease-degradable electrospun fibrous hydrogels. Nat. Commun..

[59] Highley C.B., Song K.H., Daly A.C., Burdick J.A. (2019). Jammed Microgel Inks for 3D Printing Applications. Adv. Sci..

[60] Yoshida S., Takinoue M., Iwase E., Onoe H. (2016). Dynamic transformation of self-assembled structures using anisotropic magnetized hydrogel microparticles. J. Appl. Phys..

[61] Hayakawa M., Umeyama S., Nagai K., Onoe H., Takinoue M. (2018). Controlled Construction of Stable Network Structure Composed of Honeycomb-Shaped Microhydrogels. Life.

[62] Yurke B., Turberfield A.J., Mills A.P., Simmel F.C., Neumann J.L. (2000). A DNA-fuelled molecular machine made of DNA. Nat. Cell Biol..

[63] Genot A.J., Zhang D.Y., Bath J., Turberfield A.J. (2011). Remote Toehold: A Mechanism for Flexible Control of DNA Hybridization Kinetics. J. Am. Chem. Soc..

[64] Genot A.J., Bath J., Turberfield A.J. (2012). Combinatorial Displacement of DNA Strands: Application to Matrix Multiplication and Weighted Sums. Angew. Chem. Int. Ed..

[65] Chen X. (2011). Expanding the Rule Set of DNA Circuitry with Associative Toehold Activation. J. Am. Chem. Soc..

[66] A Mechanism for Gene Conversion in Fungi|Genetics Research|Cambridge Core. https://www.cambridge.org/core/journals/genetics-research/article/mechanism-for-gene-conversion-in-fungi/E11586A6605C2A54C648BACEABECF954.

[67] Elshaarani T., Yu H., Wang L., Feng J., Li C., Zhou W., Khan A., Usman M., Amin B.U., Khan R. (2020). Chitosan reinforced hydrogels with swelling-shrinking behaviors in response to glucose concentration. Int. J. Biol. Macromol..

[68] Cangialosi A., Yoon C., Liu J., Huang Q., Guo J., Nguyen T.D., Gracias D.H., Schulman R. (2017). DNA sequence–directed shape change of photopatterned hydrogels via high-degree swelling. Science.

[69] Genot A.J., Baccouche A., Sieskind R., Aubert-Kato N., Bredeche N., Bartolo J.F., Taly V., Fujii T., Rondelez Y. (2016). High-resolution mapping of bifurcations in nonlinear biochemical circuits. Nat. Chem..

[70] Baccouche A., Okumura S., Sieskind R., Henry E., Aubert-Kato N., Bredeche N., Bartolo J.-F., Taly V., Rondelez Y., Fujii T. (2017). Massively parallel and multiparameter titration of biochemical assays with droplet microfluidics. Nat. Protoc..

[71] Sia S.K., Whitesides G.M. (2003). Microfluidic devices fabricated in Poly(dimethylsiloxane) for biological studies. Electrophoresis.

[72] Kandatsu D., Cervantes-Salguero K., Kawamata I., Hamada S., Nomura S.-I.M., Fujimoto K., Murata S. (2016). Reversible Gel-Sol Transition of a Photo-Responsive DNA Gel. ChemBioChem.

[73] Hiraide M., Ishikawa K., Kawaguchi H. (1996). Water-in-oil emulsion containing oxine for the collection of traces of copper(II) in water. Anal. Bioanal. Chem..

[74] Schmitt M., Limage S., Denoyel R., Antoni M. (2017). Effect of SPAN80 on the structure of emulsified aqueous suspensions. Colloids Surfaces A Physicochem. Eng. Asp..

[75] Xing Z., Caciagli A., Cao T., Stoev I., Zupkauskas M., O’Neill T., Wenzel T., Lamboll R., Liu D., Eiser E. (2018). Microrheology of DNA hydrogels. Proc. Natl. Acad. Sci. USA.

[76] Seeman N.C. (1982). Nucleic acid junctions and lattices. J. Theor. Biol..

[77] Cherry K.M., Qian L. (2018). Scaling up molecular pattern recognition with DNA-based winner-take-all neural networks. Nat. Cell Biol..

[78] Gaweł K., Barriet D., Sletmoen M., Stokke B.T. (2010). Responsive Hydrogels for Label-Free Signal Transduction within Biosensors. Sensors.

[79] Jiang C., Li X., Zhao H., Liu H. (2016). Long non-coding RNAs: Potential new biomarkers for predicting tumor invasion and metastasis. Mol. Cancer.

[80] Klingenberg M., Matsuda A., Diederichs S., Patel T. (2017). Non-coding RNA in hepatocellular carcinoma: Mechanisms, biomarkers and therapeutic targets. J. Hepatol..

[81] Peng Z., Liu C., Wu M. (2018). New insights into long noncoding RNAs and their roles in glioma. Mol. Cancer.

[82] Charles A., Janeway J., Travers P., Walport M., Shlomchik M.J. (2001). The Structure of a Typical Antibody Molecule. Immunobiology: The Immune System in Health and Disease.

[83] Miyata T., Asami A.N., Uragami T. (1999). Preparation of an Antigen-Sensitive Hydrogel Using Antigen−Antibody Bindings. Macromolecules.

[84] Charles P.T., Goldman E.R., Rangasammy J.G., Schauer C.L., Chen M.-S., Taitt C.R. (2004). Fabrication and characterization of 3D hydrogel microarrays to measure antigenicity and antibody functionality for biosensor applications. Biosens. Bioelectron..

[85] Miyata T., Asami N., Uragami T. (1999). A reversibly antigen-responsive hydrogel. Nat. Cell Biol..

[86] Bartosh T.J., Ylöstalo J.H., Mohammadipoor A., Bazhanov N., Coble K., Claypool K., Lee R.H., Choi H., Prockop D.J. (2010). Aggregation of human mesenchymal stromal cells (MSCs) into 3D spheroids enhances their antiinflammatory properties. Proc. Natl. Acad. Sci. USA.

[87] Tomasi R.F.-X., Sart S., Champetier T., Baroud C.N. (2020). Individual Control and Quantification of 3D Spheroids in a High-Density Microfluidic Droplet Array. Cell Rep..

[88] Sart S., Tomasi R.F.-X., Amselem G., Baroud C.N. (2017). Multiscale cytometry and regulation of 3D cell cultures on a chip. Nat. Commun..

