# Supplementary figures and images for: Morphological Manipulation of DNA Gel Microbeads with Biomolecular Stimuli
Source: Nanomaterials (Basel). 2021 Jan 22;11(2):293. doi: 10.3390/nano11020293 (PMC7912653; doi:10.3390/nano11020293)

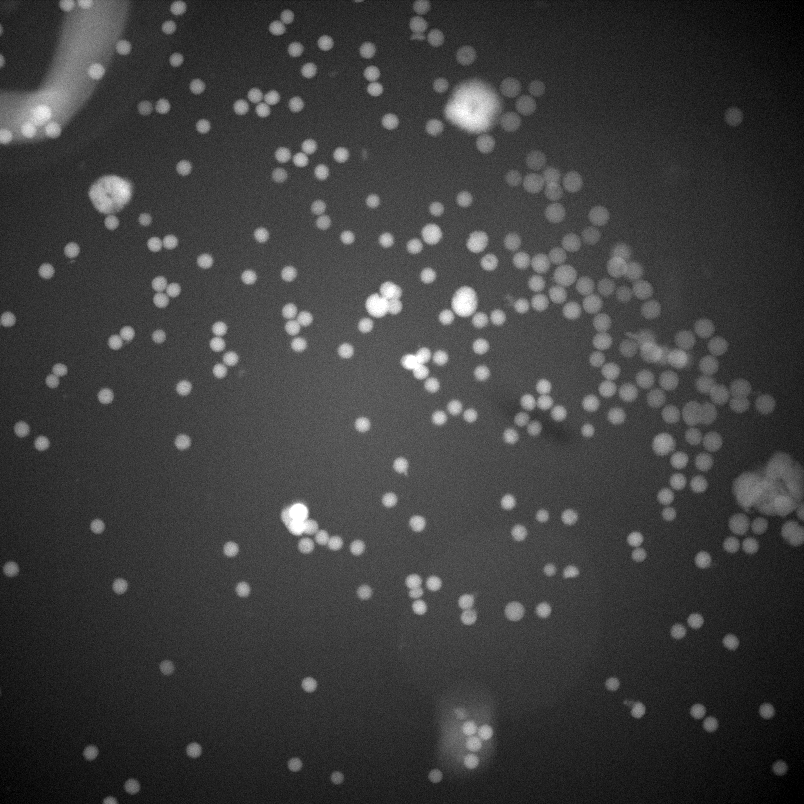

Supplement: Supplementary file 1 [file nanomaterials-11-00293-s001.zip › data/swelling dnahydrogels.tif]

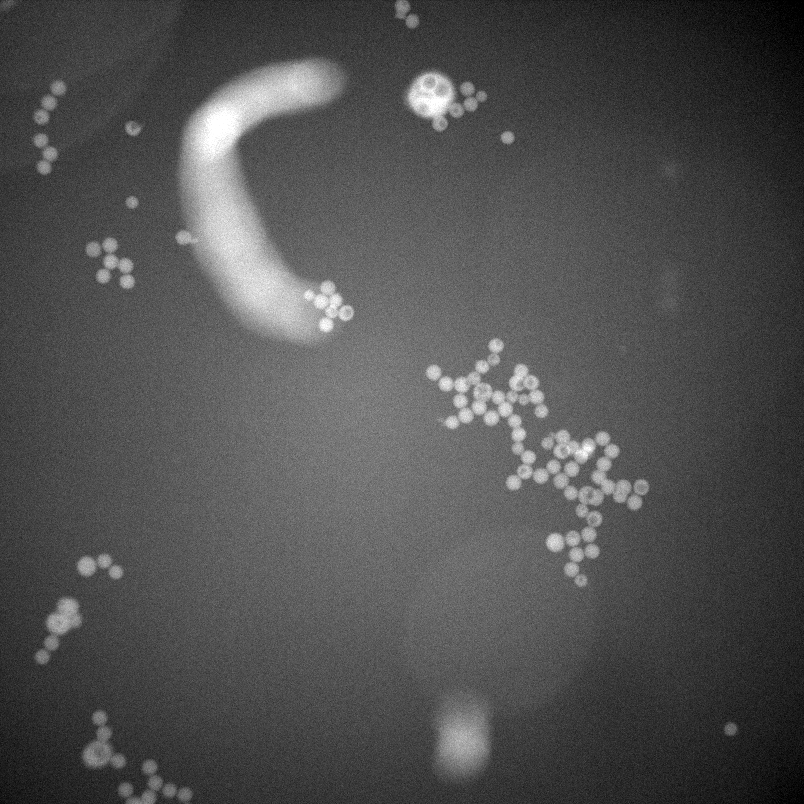

Supplement: Supplementary file 1 [file nanomaterials-11-00293-s001.zip › data/shrinking dnahydrogels.tif]
